# IRIS study: a phase II study of the steroid sulfatase inhibitor Irosustat when added to an aromatase inhibitor in ER-positive breast cancer patients

**DOI:** 10.1007/s10549-017-4328-z

**Published:** 2017-06-13

**Authors:** Carlo Palmieri, Rob C. Stein, Xinxue Liu, Emma Hudson, Hanna Nicholas, Hironobu Sasano, Fouzia Guestini, Chris Holcombe, Sophie Barrett, Laura Kenny, Sadie Reed, Adrian Lim, Larry Hayward, Sacha Howell, R. Charles Coombes, On behalf of the IRIS trial participants

**Affiliations:** 1 Department of Molecular and Clinical Cancer Medicine, University of Liverpool, Liverpool, L69 3BX UK; 2 Academic Department of Medical Oncology, Clatterbridge Cancer Centre NHS Foundation Trust, Wirral, CH63 4JY UK; 3 Department of Surgery and Cancer, Imperial College London, London, W12 0NN UK; 4 NIHR University College London Hospitals Biomedical Research Centre, London, NW1 2PG UK; 5 Department of Pathology, Tohoku University School of Medicine, Sendai, Japan; 6 Breast Unit, The Linda McCartney Centre, Royal Liverpool University Hospital, Liverpool, L7 8XP UK; 7 Beatson West of Scotland Cancer Centre, Glasgow, G12 0YN UK; 8 Department of Radiology, Imperial College NHS Foundation Trust, London, W8 6RF UK; 9 Edinburgh Cancer Centre, Western General Hospital, Edinburgh, EH4 2XU UK; 10 Department of Medical Oncology, The University of Manchester, The Christie NHS Foundation Trust, Manchester, M20 4BX UK

**Keywords:** Breast cancer, Endocrine therapy, Sulfatase, Aromatase

## Abstract

**Purpose:**

Irosustat is a first-generation, orally active, irreversible steroid sulfatase inhibitor. We performed a multicentre, open label phase II trial of the addition of Irosustat to a first-line aromatase inhibitor (AI) in patients with advanced BC to evaluate the safety of the combination and to test the hypothesis that the addition of Irosustat to AI may further suppress estradiol levels and result in clinical benefit.

**Experimental design:**

Postmenopausal women with ER-positive locally advanced or metastatic breast cancer who had derived clinical benefit from a first-line AI and who subsequently progressed were enrolled. The first-line AI was continued and Irosustat (40 mg orally daily) added. The primary endpoint was clinical benefit rate (CBR). Secondary endpoints included safety, tolerability, and pharmacodynamic end points.

**Results:**

Twenty-seven women were recruited, four discontinued treatment without response assessment. Based on local reporting, the CBR was 18.5% (95% CI 6.3–38.1%) on an intent to treat basis, increasing to 21.7% (95% CI 7.4–43.7%) by per-protocol analysis. In those patients that achieved clinical benefit (*n* = 5), the median (interquartile range) duration was 9.4 months (8.1–11.3) months. The median progression-free survival time was 2.7 months (95% CI 2.5–4.6) in both the ITT and per-protocol analyses. The most frequently reported grade 3/4 toxicities were dry skin (28%), nausea (13%), fatigue (13%), diarrhoea (8%), headache (7%), anorexia (7%) and lethargy (7%).

**Conclusions:**

The addition of Irosustat to aromatase inhibitor therapy resulted in clinical benefit with an acceptable safety profile. The study met its pre-defined success criterion by both local and central radiological assessments.

**Electronic supplementary material:**

The online version of this article (doi:10.1007/s10549-017-4328-z) contains supplementary material, which is available to authorized users.

## Introduction

Inhibition of the activity of the transcription factor oestrogen receptor alpha (ER) represents a cornerstone strategy in the management of ER-positive (ER+) breast cancer (BC). Lowering of circulating estradiol levels through the inhibition of peripheral aromatase enzyme activity is one of the key endocrine manipulations used in the management of postmenopausal BC [1, 2]. However, the second major pathway for oestrogen biosynthesis, steroid sulfatase (STS), is yet to be exploited therapeutically.

STS is responsible for the hydrolysis of steroid sulfates to their unconjugated, biologically active forms converting estrone sulphate (E1S) and DHEAS to estrone and DHEA, respectively. STS mRNA is expressed in the majority of ER+ breast tumours and is inversely associated with survival [3, 4]. Expression of STS protein has been reported in 74% of breast cancers and its expression is significantly associated with larger tumour size, and with an increased risk of recurrence and poorer overall survival [5]. Conversely, the expression of oestrogen sulfotransferase (EST), which opposes the actions of STS, inversely correlates with tumour size, lymph node status and is significantly associated with a decreased risk of recurrence and improved overall survival [5]. The importance of DHEAS as a precursor for androstenediol was shown by its ability to stimulate the proliferation of breast cancer cells, which could be blocked with an anti-oestrogen or STS inhibitor, but not an AI [6]. Serum DHEAS levels have been shown to be significantly elevated in women progressing on an AI [7] suggesting that androstenediol production from DHEAS may be a mechanism of AI resistance. This is supported by data from a neoadjuvant study, which found an increase in STS following exposure to an aromatase inhibitor (AI) [8]. This could therefore represent a compensatory and adaptive response to the blockade of aromatase and the subsequent depletion of intratumoral oestrogen.

Irosustat (STX64) a tricyclic coumarin sulphamate is a first-generation irreversible inhibitor of STS [9]. Two phase I studies of Irosustat have been performed [10, 11]. In the first, 14 women who had progressed on two prior lines of endocrine therapy (ET) were treated with either 5 or 20 mg doses of Irosustat [10]. STS activity as measured in peripheral blood lymphocytes (PBLs) and within the breast tumours was almost completely inhibited following treatment with Irosustat. As predicted by its mechanism of action, there was a significant suppression of serum estrone, estradiol, androstenediol and DHEA [10]. Four patients (all previously progressed on AI) had stable disease for 2.75–7 months. In the second study, performed following reformulation, the optimal biological dose (OBD) was found to be 40 mg daily based on the reduction of STS activity in peripheral blood, changes in circulating steroidogenic hormones and the lack of grade 3 toxicity in the first 28 days [11]. No objective responses were seen, and the median time to progression for the 40 mg group was 10.1 [(3.0–72.3) weeks]. Disease stabilisation was seen in three of thirteen patients at the 40 mg dose, all of whom remained progression free for at least 24 weeks (range 27.1–72.3 weeks). An FDG-PET-scan sub-study carried out in six patients at the 40 mg dose revealed that 50% of patients displayed significant median decreases in standardised uptake mean value (SUVmean), and hypermetabolic tissular volume (HT Volume) at Day 28 [11]. In both studies, Irosustat was well tolerated with no biochemical or haematologic toxicities [10, 11]. Based on the safety profile of Irosustat from these initial phase I studies and the known side effect profile of AIs, no safety issues were expected with regard to combining Irosustat with an AI. Therefore, a phase II combination study was developed.

The IRIS study was designed to investigate the efficacy and tolerability of Irosustat in postmenopausal women who had progressed on a first-line aromatase inhibitor (AI) from which they had derived clinical benefit. This study aimed to test the hypothesis that the blockade of STS with Irosustat on the background of continued aromatase inhibition could result in clinical benefit. Safety and pharmacodynamic endpoints were also assessed.

## Materials and methods

### Study design

The IRIS study (ClinicalTrials.gov identifier NCT01785992) was a multicentre, open label phase II trial performed in nine academic medical centres in the United Kingdom (full list in supplementary information). Ethical approval was given by the NRES Committee London-Riverside (an Independent Ethics Committee; reference 12/LO/0477), as well as being approved by the United Kingdom Medicines and Healthcare Products Regulatory Agency (EudraCT: 2011-005680-25). All patients provided written informed consent.

## Eligibility

Women were eligible if they were postmenopausal, with histologically confirmed ER-positive, HER-2 negative inoperable locally advanced or metastatic breast cancer. ER positivity was based on local laboratory assessment. Patients had to have developed progressive disease during first-line AI treatment for recurrent ER-positive breast cancer. Furthermore, patients had to have derived clinical benefit, defined as a documented objective response at any point or disease stabilisation (SD) for at least 6 months, from this first-line AI treatment. The disease had to be measurable by CT/MRI scan according to RECIST v1.1. (Full inclusion and exclusion criteria in supplementary information).

### Trial treatment

Irosustat was given orally at a dose of 40 mg daily in addition to the first-line AI, which was continued beyond progression. No other therapy was given in the intervening time between progression on the AI and commencement of Irosustat. Combined therapy was continued until disease progression, death, the development of unacceptable toxicities or the withdrawal of consent.

### Trial assessments

Clinical assessments and toxicity reporting were performed every month (28 days) for the first 6 months and 3 monthly thereafter until disease progression, occurrence of unacceptable toxicities or withdrawal from treatment. A safety visit was performed 7 days after Irosustat was discontinued. Tumour response was evaluated every 3 months according to RECIST version 1.1. Adverse events (AEs) were graded according to National Cancer Institute Common Terminology Criteria (version 4.3), with relationship to study medication recorded, and coded using the Medical Dictionary for Regulatory Activities (MedDRA version 14.0). Formalin-fixed paraffin-embedded (FFPE) samples were requested of the primary tumour and any recurrence or metastatic site that had been biopsied prior to study entry. Blood samples were collected and processed at baseline, every month and on progression (see supplementary information).

### Study endpoints

The primary endpoint was clinical benefit rate which was defined as the proportion of patients with either complete response (CR) or partial response (PR) at any scheduled tumour assessment, or stable disease (SD) for at least 6 months. Secondary endpoints included progression-free survival (PFS), defined as time from study enrolment to first evidence of PD or death due to any cause, duration of clinical benefit as defined by the number of days from start of study drug to the first evidence of progressive disease (PD) or death due to any cause; objective response rate defined by the proportion of CR and PR. Safety and tolerability as assessed by the collection of adverse events (AE) according to the Common Terminology Criteria for Adverse Events (NCI-CTCAE v 4.03) and to measure alterations in circulating steroid hormones and correlate these measures with clinical outcome. Exploratory translational endpoints included the assessment of the expression of steroidogenic enzymes, i.e. STS, aromatase, EST, 17βHSD1 and 17βHSD2. Central review of all study imaging was undertaken by an independent radiologist.

### Steroidogenic hormone profiling

Steroidogenic Hormone profiling was carried out by a central laboratory, Quest Diagnostics (Nichols Institute, San Juan Capistrano, CA, USA). Androstenedione, oestrone sulfate (E1S), dehydroepiandrosterone sulphate (DHEAS), dehydroepiandrosterone (DHEA), androstenediol and testosterone were quantitated using a TSQ Quantum Ultra (Thermo Fisher; San Jose, CA) triple quadrupole tandem mass spectrometer. Estrone and estradiol were detected and quantitated in negative ionisation mode using a triple quadrupole tandem mass spectrometer with APCI source (TSQ Quantum Ultra, Thermo Fisher; San Jose, CA). Further detailed methodology is provided in the supplementary materials and methods.

### Immunohistochemistry staining

Immunohistochemistry was performed for the expression of four enzymes involved in oestrogen metabolism (aromatase, steroid sulfatase, oestrogen sulfotransferase, 17beta-Hydroxysteroid dehydrogenase type 1 and type 2); detailed methodology is described in supplementary materials and methods.

Immunostained slides were independently evaluated by two of the authors (FG, HS) who are experienced in scoring the four biomarkers concerned, both were blinded to patients’ clinical outcomes. Marker’s expression was evaluated by assigning scores for the approximate percentage of immunopositive cells (proportion score) and for staining intensity, which were added together. The range of proportion scores for aromatase was 0–3 as follows: 0 ≤ 1%, 1 = 1–25%, 2 = 26–50% and 3 ≥ 50% immunopositive cells (Sasano et al. 2009). The range of proportion score for STS, EST and 17BHSD was 0–2 as follows: 0 = no stained tumour cells, 1 = 1–50%, 2 ≥ 50% immunopositive cells (5). Relative staining intensity of immunopositive cells was classified as 0 = no immunoreactivity, 1 = weak, 2 = moderate and 3 = strong immunoreactivity.

### Statistical analysis

The study used a Simon’s minimax two-stage design to provide 80% power with a one-sided type I error of 0.05 to declare the treatment effective assuming a maximum unacceptable CBR (p0) of 5% and a minimum acceptable CBR (p1) of 20%. In the first stage, 13 patients were to be evaluated, and if at least one patient achieved clinical benefit, another 14 patients would be enrolled. If the clinical benefit was seen in 4 out of 27 patients overall, the treatment would be declared effective. For PFS, patients were censored at the time of the last follow-up if they had withdrawn or been lost to follow-up before progression or death. PFS was estimated using the Kaplan–Meier method. All 27 patients enrolled in the study and who received at least one dose of drug, formed the Intent to treat population, while the per-protocol group included all patients except three patients who withdraw themselves and one who was withdrawn by the local principal investigator prior to the first tumour assessment at 3 months.

## Results

**Fig. 1:**
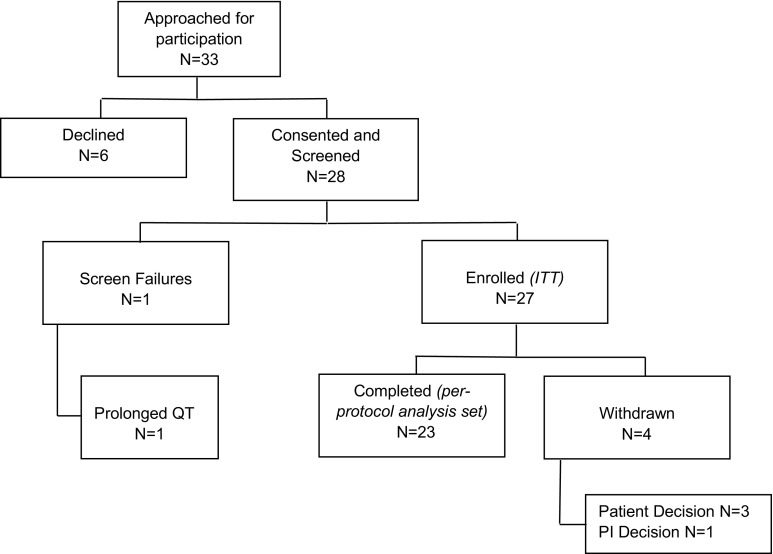
CONSORT trial diagram. *ITT* intent to treat, *PI* principal investigator

Between February 2013 and March 2014 28 patients were consented. One patient was found to be ineligible prior to starting treatment. All 27 patients enrolled in the study received at least one dose of drug, and formed the ITT population. Four patients withdrew from the study before the first tumour assessment (Fig. 1). The baseline clinicopathological details of patients are provided in Table 1. Prior to recruitment, all 27 patients were receiving an AI as first-line therapy with a median (IQR) duration of treatment of 21.1 (13.3–37.6) months; 27% of patients had received one course of chemotherapy for advanced disease.

**Table 1:** Clinicopathological details

	ITT *N* = 27	Per-protocol analysis *N* = 23
Age (years)	63.7 (10.5)	63.3 (9.9)
BMI (kg/m^2^)	28.1 (6.3)	28.2 (6.6)
Duration of the AI treatment at enrolment (month)^a^	21.1 (13.3–37.6)	21.1 (10.3–35.7)
Time between primary diagnosis to metastatic diagnosis (month)^a^	60.7 (2.0–116.1) [*n* = 23]	60.7 (2.0–105.4) [*n* = 19]
Ethnicity		
White	24 (88.9%)	21 (91.3%)
Asian	1 (3.7%)	1 (4.4%)
Black	1 (3.7%)	0 (0%)
Chinese	1 (3.7%)	1 (4.4%)
No. of sites of disease		
1	7 (25.9%)	6 (26.1%)
2	9 (33.3%)	8 (34.8%)
3	7 (25.9%)	7 (30.4%)
4	1 (3.7%)	0 (0%)
5	2 (7.4%)	1 (4.4%)
Missing	1 (3.7%)	1 (4.4%)
Visceral disease		
No	6 (22.2%)	6 (26.1%)
Yes	20 (74.1%)	16 (69.6%)
Missing	1 (3.7%)	1 (4.4%)
ER status^b^		
Positive	27 (100%)	23 (100%)
Negative	0 (0%)	0(0%)
PgR status^b^		
Positive	20 (74.1%)	16 (69.6%)
Negative	4 (14.8%)	4 (17.4%)
Unknown	3 (11.1%)	3 (13.0%)
HER2 status^b^		
0	10 (37.0%)	10 (43.5%)
1+	10 (37.0%)	7 (30.4%)
2+	3 (11.1%)	2 (8.7%)
Amplified	0 (0%)	0 (0%)
3+^c^	1 (3.7%)	1 (4.4%)
Not done	3 (11.1%)	3 (13.0%)
Treatment history: chemotherapy		
No	10 (37.0%)	9 (39.1%)
Yes	17 (63.0%)	14 (60.9%)
Neoadjuvant	2 (9.1%)	1 (5.9%)
Adjuvant	14 (63.6%)	11 (64.7%)
Advanced 1st line	6 (27.3%)	5 (29.4%)
Treatment history: radiotherapy		
No	13 (48.1%)	11 (47.8%)
Yes	14 (51.9%)	12 (52.2%)
Adjuvant	19 (63.3%)	18 (66.7%)
Palliative	11 (36.7%)	9 (33.3%)
Treatment history: endocrine therapy		
No	0 (0%)	0 (0%)
Yes	27 (100.0%)	23 (100.0%)
Neoadjuvant		
Anastrozole	1 (2.1%)	1 (2.5%)
Adjuvant		
Exemestane	1 (2.1%)	1 (2.5%)
Letrozole	1 (2.1%)	1 (2.5%)
Anastrozole	4 (8.5%)	3 (7.5%)
Tamoxifen	16 (34.0%)	13 (32.5%)
Advanced 1st line		
Exemestane	3 (6.4%)	3 (7.5%)
Letrozole	17 (36.2%)	14 (35.0%)
Anastrozole	4 (8.5%)	4 (10.0%)

*ITT* intent to treat

Data presented are mean (SD) for continuous variables and frequency (percentage) for categorical variables

^a^Data presented are median (inter quartile range)

^b^Based on diagnostic biopsy and primary tumour sample

^c^HER2 results: 3+ on diagnostic biopsy and 1+ on resected tumour

Drug compliance was monitored using a patient diary. Of the 27 patients in the study, drug compliance data were available for 26 patients as one patient mislaid her drug diary. The compliance with both AI treatment and Irosustat during the study was very good with median rates of 100% (range 90.5–100%) and 100% (range 87.0–100%), respectively.

## Efficacy

For the 27 patients recruited, the median duration of treatment was 2.8 months (range 1.5–17.4 months). Based on the local study sites’ tumour assessment, there were no objective responses. At the interim analysis when the primary outcome for the first 13 patients was available, we observed three patients with clinical benefit (three stable diseases for at least 6 months) and the trial moved to the second stage, where another two patients with clinical benefit were observed. In summary, five patients had stable disease for at least 6 months giving a clinical benefit rate (CBR) of 18.5% (95% CI 6.3–38.1%) on an intent to treat basis (Table 2). In a per-protocol analysis which excluded the four patients who withdrew before the first tumour assessment, the CBR was 21.7% (95% CI 7.4–43.7%).

**Table 2:** Efficacy analysis on the basis of local and central assessment

	ITT [*n* = 27]	Per protocol [*n* = 23]
*Local assessment*		
Clinical benefit rate	18.5% (6.3–38.1%)	21.7% (7.4–43.7%)
Response at 6 month scan		
Complete response	0	0
Partial response	0	0
Stable disease	5	5
Progressive disease	16	16
Dead	1	1
Withdrawal	5	1
*Central assessment*		
Clinical benefit rate	14.8% (4.2–33.7%)	17.4% (5.0–38.8%)
Response at 6 month scan		
Complete response	0	0
Partial response	0	0
Stable disease	4	4
Progressive disease	15	15
Dead	1	1
Withdrawal^a^	7	3

*ITT* intent to treat; Prior to 3 month scan: three patients decided to withdraw; one patient withdrawn by local investigator. These four patients had no scan and were excluded in the per-protocol analysis; Between 3 and 6 month scan: one patient withdrawn

^a^At month 3: two patients PD reclassified as SD by central review and thus treated as withdrawals

Central review was undertaken for all patients where at least one radiological assessment had been undertaken (i.e. the per-protocol group) which confirmed that there were no objective responses (Table 2). As a result of the central review, four patients derived clinical benefit, giving a CBR of 14.8% (95% CI 4.2–33.7%) based on intent to treat analysis and 17.4% (95% CI 5.0–38.8%) in the per-protocol analysis.

**Fig. 2:**
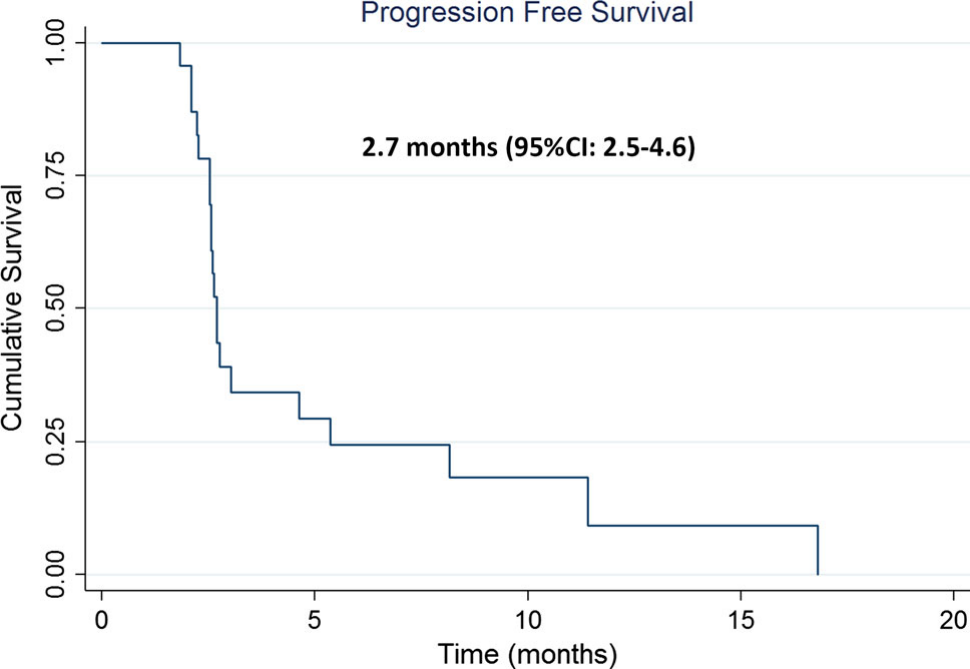
Progression-free survival as assessed by the investigators in the intention-to-treat population

The median (IQR) duration of clinical benefit in the local ITT analysis of 26 patients was 9.4 months (8.1–11.3); the precise date of starting study drug was unknown for one patient. PFS based on local review was 2.7 months (95% CI 2.5–4.6) in both the ITT and the per-protocol analysis (Fig. 2).

## Adverse events

All twenty-seven patients experienced treatment-emergent adverse events, 91% of which were grade 1 or 2 (Table 3). The most common were dry skin (77%), nausea (48%) and fatigue (40%). Grade 2 ECG abnormalities (QT prolongation) were reported in one patient, which were considered unrelated to study drug. Three patients were discontinued due to AEs; these were urinary tract infection, possible renal toxicity (not reported as AE) and dry skin.

**Table 3:** Treatment-emergent adverse events regardless of relationship to study drugs

Adverse event	Grade 1–2	Grade 3–4	Grade 5
Dry skin	21	1	
Nausea	13	2	
Fatigue	11	2	
Diarrhoea	8	0	
Decreased appetite	5	0	
Headache	5	0	
Lethargy	5	0	
Vomiting	4	0	
Rash	3	1	
Cough	3	0	
Dizziness	3	1	
Arthralgia	3	0	
Insomnia	3	0	
Sepsis	0	1	
Haemoglobin	0	1	
Urinary tract infection	0	1	
Breast ulceration	0	1	
Gamma GT increase	0	2	
Blurred vision	0	1	
Bone pain	0	1	
Pleurodesis	0	1	
Pneumonia	0	0	1

Grades 1 and 2 with an incidence of 10% of study population and all grade 3–5 toxicities

Nine serious adverse events occurred in 6 patients (22%). None were considered definitely related to study drug; one was considered probably related (nausea and vomiting) and another possibly related (nausea and vomiting). All other SAEs were considered as either unlikely to be related (sepsis, urinary tract infection, breast pain) or not related (cellulitis secondary to an animal bite, symptoms of morphine toxicity, pneumonia). There was one death due to bronchopneumonia during the study that was unrelated to the study medication.

## Pharmacodynamic data

At study entry and at all subsequent monthly time points, the circulating levels of estradiol and estrone were below the threshold of detection in all patients (2 and 10 ng/dl, respectively, data not show). There were significant decreases in androstenedione, DHEA (*p* < 0.01) and testosterone (*p* = 0.03), as well as significant increases in DHEAS (*p* = 0.02) and DHEA:DHEAS ratio (*p* < 0.01) at 3 months (Table 4). At 6 months with fewer samples, available statistical significance was lost.

**Table 4:** Changes in circulating steroid hormones

	Baseline [*N* = 27]	1 month [*N* = 27]	2 months [*N* = 22]	3 months [*N* = 20]	4 months [*N* = 8]	5 months [*N* = 7]	6 months [*N* = 6]	*P* for 3 months^a^	*P* for 6 months^a^
Androstenedione (ng/dl)	59.5 (46.8–78.2) [*n* = 24]	35.0 (28.5–55.0) [*n* = 23]	44.0 (41.0–63.0) [*n* = 21]	46.0 (30.5–55.5) [*n* = 19]	36.0 (30.5–52.0) [*n* = 7]	23.0 (21.0–29.0) [*n* = 7]	36.0 (31.5–42.0) [*n* = 4]	<0.01 [*n* = 17]	0.29 [*n* = 3]
Oestrone sulfate (ng/dl)	40.0 (40.0– 70.8) [*n* = 26]	40.0 (40.0– 70.0) [*n* = 26]	40.0 (40.0– 81.0) [*n* = 21]	40.0 (40.0– 53.5) [*n* = 20	40.0 (40.0–158.0) [*n* = 7]	65.0 (40.0–152.5) [*n* = 7]	61.0 (57.0–113.8) [*n* = 6]	0.53 [*n* = 20]	0.53 [*n* = 6]
DHEA (ng/dl)	118.5 (77.5–194.0) [*n* = 24]	84.5 (56.0–137.2) [*n* = 24]	83.0 (43.0–155.0) [*n* = 21]	62.5 (47.8–105.8) [*n* = 20]	56.0 (40.5– 73.0) [*n* = 7]	69.0 (48.0– 75.0) [*n* = 7]	53.0 (26.8– 77.8) [*n* = 6]	<0.01 [*n* = 18]	0.07 [*n* = 4]
DHEAS (ng/dl)	64.0 (51.0– 98.5) [*n* = 23]	149.5 (104.0–196.8) [*n* = 22]	135.0 (73.5–175.0) [*n* = 19]	146.0 (46.0–188.0) [*n* = 17]	84.0 (76.0–126.5) [*n* = 7]	103.0 (81.2–151.8) [*n* = 4]	124.0 (74.0–187.0) [*n* = 5]	0.02 [*n* = 16]	0.14 [*n* = 4]
Testosterone (ng/dl)	12.0 (8.0–21.0) [*n* = 21]	10.5 (8.8–15.2) [*n* = 16]	11.0 (6.0–15.0) [*n* = 17]	11.5 (6.2–17.2) [*n* = 14]	8.5 (6.0–12.5) [*n* = 6]	8.0 (3.5–12.2) [*n* = 4]	5.0 (4.0–10.0) [*n* = 5]	0.03 [*n* = 12]	0.07 [*n* = 4]
DHEA: DHEAS ratio	1.9 (1.2–2.5) [*n* = 23]	0.7 (0.4–1.3) [*n* = 21]	0.9 (0.5–1.0) [*n* = 19]	0.7 (0.5–1.0) [*n* = 17]	0.6 (0.3–1.0) [*n* = 7]	0.7 (0.3–0.9) [*n* = 4]	0.4 (0.3–0.9) [*n* = 5]	<0.01 [*n* = 16]	0.07 [*n* = 4]

*DHEA* dehydroepiandrosterone, *DHEAS* dehydroepiandrosterone sulphate

Data presented are median (inter quartile range)

^a^ *P* value for Wilcoxon signed rank test comparing baseline and 3-month/6-month follow-up

## Immunohistochemistry data

STS, EST, Aromatase, 17BHSD1 and 17BHSD2 immunostaining was successfully performed on the primary tumours from 19 patients, a biopsy of the first relapse in three cases and on AI progression in one cases and includes three who derived clinical benefit from Irosustat. (Supplementary Table 1 and representative micrographs of staining in supplementary Fig. 1). Of note, only 37% (7 of 19) and 25% (1 of 4) of primary and recurrent samples had moderate to strong (score ≥4) positivity for STS.

## Discussion

IRIS is the first study to explore the efficacy of the sulfatase inhibitor Irosustat in ER-positive metastatic breast cancer in combination with an AI. In this study, we added steroid sulfatase inhibition to the background aromatase inhibition upon which disease progression had occurred. The underlying rationale for the study is based on multiple observations. Firstly, that after estrone is synthesised from androstenedione (Adione), much of it is rapidly sulphated to estrone sulfate (E1S), by the enzyme oestrogen sulfotransferase (EST) [12]. E1S is known to have a plasma concentration 10–20 times higher than those of the unconjugated oestrogens estrone and estradiol, as well as a longer half-life in the plasma than the unconjugated oestrogens and therefore acts as a reservoir for active estrogens [13]. E1S uptake into malignant breast tissue is facilitated by organic anion transporter polypeptide B (OATP) [14]. Secondly, androgens derived from the adrenal cortex are known to have oestrogenic effects [15]. Thirdly, STS, the enzyme responsible for the hydrolysis of steroid sulphates to their unconjugated biologically active forms, is overexpressed in breast cancer and is known to be associated with a worse outcome [4, 5]. Fourthly, STS is upregulated following exposure to an aromatase inhibitor (AI) which is a putative mechanism of resistance to AIs [8]. Fifthly, the additive use of oestrogen synthesis inhibition can result in clinical benefit as seen when an AI is added to ovarian suppression in premenopausal women with breast cancer [16]. We therefore hypothesised that the addition of an STS inhibitor to an AI at disease progression would result in a lowering of peripheral and intratumoral estrone and DHEA, reversal of resistance and clinical benefit.

The principal findings relate to effect on disease status, adverse events and effects on steroid hormones. Firstly, regarding the anti-tumour effects of Irosustat, the study met its primary endpoint of showing evidence of clinical benefit in a second-line setting based on both local and central reviews, with a median PFS of 2.7 months. Although there were no objective responses, a number of patients experienced disease stabilisation for at least 6 months. It is known that the objective response and clinical benefit rates of second-line endocrine treatment are limited. For example, the objective response rate to second-line endocrine therapy in large phase III studies with exemestane or high-dose fulvestrant has been reported to be in the range of 0.4–6.3%, while median PFS in these studies ranged from 2.8 to 3.8 months [17–19]. Therefore, Irosustat as a single agent added onto the background of an AI has evidence of clinical activity and, with the caveats relating to cross trial comparison and their limitations, does show efficacy which is comparable to both exemestane and high-dose fulvestrant in this setting. Future studies need to explore the clinical activity of dual aromatase and STS blockade as first-line metastatic treatment in a patients likely to be endocrine sensitive, as well selecting for STS expression.

The suppression of circulating estradiol is a key therapeutic strategy in the management of ER-positive breast cancer, and all the women who entered this study had estrone and estradiol levels below the level of detection as the result of AI treatment. However, steroidogenic hormones derived from the adrenal gland have been implicated in AI resistance. DHEAS is the most abundant steroid in the human circulation and has been shown to be significantly elevated in women whose disease failed to respond to an AI in the metastatic setting [7]. DHEA has been shown to stimulate the proliferation of the oestrogen-dependent MCF-7 human breast cancer cell line, as well as a derived oestrogen-independent variant, and to transactivate ER in both lines [20]. In vitro DHEAS has been shown to transactivate both ER and the AR in a dose-dependent manner, with the DHEAS induced AR transactivity abolished by Irosustat [21]. DHEA can itself be converted to androstenediol by 17β-HSD1, as well as to androstenedione via 3β-HSD1 and 17β-HSD2. Androstenediol is known to have potent oestrogenic effects [15], and can stimulate the growth of hormone-dependent breast cancer cells both in vitro and in vivo [22, 23]. Androstenedione has been demonstrated to induce recruitment of ER and SRC3 to gene promoters and drive ER transcription [24] and AI resistance in vitro [25]. Androstenedione was significantly elevated in women on a second-line AI for metastatic breast cancer as compared to women being treated in the adjuvant setting [26]. Furthermore, androstenedione can be converted to 5a-androstane-3b, 17b–diol (3bAdiol) which is itself estrogenic, and can induce growth in breast cancer cells [27]. This compound has also been implicated in endocrine resistance [28]. Clinically, previous studies have also shown that AIs do not affect the levels of androstenediol [29], while serum DHEAS was significantly higher in women who progressed on an AI [7]. Steroids with potent estrogenic properties may provide an escape mechanism for growth within a low estradiol milieu.

Steroidogenic hormone measurements within the current study revealed treatment with Irosustat resulted in significant and predicable rise at 3 months in estrone sulphate and DHEAS, and decrease in DHEA:DHEAS as well as significant reduction in androstenedione at 3 months. These pharmacodynamics data confirm that Irosustat had effected sulfatase inhibition in our patients. Expression of STS and other enzymes involved in oestrogen biosynthesis were assessed predominately in primary breast cancer samples. Given the small numbers, no formal assessment can be made of the possible use of STS as a biomarker of response. However, moderate to strong expression of STS was observed in only 37 and 25% of the primary and recurrent cases, and the preponderance of tumours with low STS expression clearly may have impacted on the efficacy data. Furthermore, the primary tissue was mainly used and this may not reflect the biology of a tumour which has progressed following a prolonged period of oestrogen deprivation. Ideally, in future studies of Irosustat a metastatic biopsy should be mandated prior to study entry [30], and prospectively enrich for a STS high population of tumours.

Irosustat was well tolerated with most adverse effects being grades 1 and 2, and the most common being dry skin, nausea and fatigue. Dry skin is an expected side effect being reported in both phase I studies [10, 11]. This is expected given that hereditary deficiency of STS results in dry, scaly skin as a result of abnormal corneocyte retention and thickening of the stratum corneum [31].

There are several limitations to our study. Primary tumours were utilised for the IHC assessment since metastatic biopsies from the time of study entry were not routinely collected [30], particularly given the evidence that STS is upregulated following exposure to an AI [8]. Since the study was initiated, the development of ESR1 mutations as a resistance mechanism to AIs has been described. As all patients who entered the study had progressed on an AI, it would be expected that 20–30% would harbour ESR1 mutations [32–34]. The possible effect of ESR1 mutations with regard to the efficacy of Irosustat is unknown. The study strengths are that we undertook local and central review of radiology, with the study meeting its primary endpoint by both assessments.

More recently, it has been demonstrated that the addition of either an mTOR inhibitor or a CDK4/6 inhibitor to an AI can significantly improve the outcomes of women with ER-positive metastatic breast cancer [17, 35]. Given the clinical activity of these targeted therapies is dependent on the dual targeting of ER with endocrine therapy, the development of a more effective ET backbone may lead to enhance efficacy of these biological therapies. Therefore, any future clinical development will need to attempt to address the question of whether an ET backbone which comprises it and an AI is more clinically effective than an AI alone when combined with targeted therapies such an mTOR or CDK4/6 inhibitors.

In summary, this proof of concept study provides evidence for the first time that combining both an AI and a STS inhibitor can have clinical activity and that the combination is safe and well tolerated. Future studies should explore the clinical activity of Irosustat at an early line of treatment in a populations enriched for STS expression.

## Electronic supplementary material

Below is the link to the electronic supplementary material.

Supplementary material 1 (DOCX 16 kb)

Supplementary material 2 (DOCX 18 kb)

Supplementary material 3 (DOCX 19 kb)

Supplementary material 4 (PPTX 22879 kb)
