# Supplementary material for: IRIS study: a phase II study of the steroid sulfatase inhibitor Irosustat when added to an aromatase inhibitor in ER-positive breast cancer patients
Source: Breast Cancer Res Treat. 2017 Jun 13;165(2):343–53. doi: 10.1007/s10549-017-4328-z (PMC5543190; doi:10.1007/s10549-017-4328-z)
Supplement: Supplementary file 1 — Supplementary material 1 (DOCX 16 kb) [file 10549_2017_4328_MOESM1_ESM.docx]

**Supplementary Information**

**Participating Centres**

There were nine participating centres across the UK: which are listed below

• IRIS001: Charing Cross Hospital, Imperial College Healthcare NHS Trust, UK;

• IRIS002: The Beatson West of Scotland Cancer Centre, NHS Greater Glasgow  and Clyde, UK;

• IRIS003: Western General Hospital, NHS Lothian, UK;

• IRIS004: University College London NHS Foundation Trust, UK;

• IRIS005: West Middlesex University Hospital, Chelsea and Westminster Hospital  NHS Foundation Trust, UK;

• IRIS006: The Christie NHS Foundation Trust, UK;

• IRIS008: Broomfield Hospital, Mid Essex Hospital Services NHS Trust, UK;

• IRIS009: Royal Free London NHS Foundation Trust, UK;

• IRIS010: The Royal Liverpool and Broadgreen University Hospitals NHS Trust, UK

**Inclusion Criteria**

1. Written informed consent prior to admission to this study.

2. Aged ≥ 25 years of age.

3. Histologically confirmed ER+ve primary or metastatic breast cancer according to local criteria.

4. Locally advanced1 or metastatic breast cancer treated with 1st line AI treatment with either:

A documented objective response (CR/PR) at any point after beginning on the 1st line AI prior to disease progression.

OR

Disease stabilisation (SD) on the 1st line AI for at least 6 months prior to dis-ease progression.

5. Postmenopausal.

6. ECOG performance status 0 to 2.

7. Measurable and/or evaluable sites of locally advanced or metastatic disease that can be accurately assessed by CT/MRI scan at baseline and follow up visits2 (RECIST v1.1).

8. Adequate haematological, hepatic and renal function.

9. Life expectancy of >3 months

NOTE: Patients on established bisphosphonate treatment for at least 3 months are eligible for entry into the trial and are allowed to continue with bisphosphonate treatment.

**Exclusion Criteria**

1. HER2 positive cancer

2. Discontinuation of current AI therapy for > 21 days prior to study entry3

3. Rapidly progressive, life-threatening metastases, including any of the following:

a. Patients with active parenchymal brain or leptomeningeal involvement.

b. Symptomatic lymphangitis carcinomatosis

c. Extensive visceral metastases requiring chemotherapy

4. Patients with a history of another primary malignancy within 5 years prior to starting study treatment, except adequately treated basal or squamous cell carcinoma of the skin, carcinoma in situ and the disease under study.

5. More than one prior chemotherapy for locally advanced or metastatic disease.

6. AI therapy given in combination with another endocrine agent with the exception of a GnRH agonist.

7. Radiotherapy to measurable lesion within 2 months of treatment start.

8. Systemic corticosteroids for > 15 days within the last 4 weeks.

9. Evidence of uncontrolled active infection.

10. Evidence of significant medical condition or laboratory finding which, in the opinion of the Investigator, makes it undesirable for the patient to participate in the trial.

11. Concurrent therapy with any other investigational agent.

12. Concomitant use of CYP2C and 3A inducers & systemic carbonic anhydrase in-hibitors.

13. Any of the following cardiac criteria:

a. Mean resting corrected QT interval (QTcf) >450 ms obtained from 3 elec-trocardiograms (ECGs)

b. Any clinically important abnormalities in rhythm, conduction or mor-phology of resting ECG e.g. complete left bundle branch block, third de-gree heart block

c. Any factors that increase the risk of QTc prolongation or risk of arrhyth-mic events such as heart failure, hypokalaemia, congenital long QT syn-drome, family history of long QT syndrome or unexplained sudden death under 40 years of age or any concomitant medication known to prolong the QT interval

14. Uncontrolled abnormalities of serum potassium, sodium, calcium or magnesi-um levels.

15. Refractory nausea and vomiting, chronic gastrointestinal diseases, inability to swallow the formulated IMP or previous significant bowel resection that would preclude absorption of Irosustat or the AIs.
