# Supplementary material for: IRIS study: a phase II study of the steroid sulfatase inhibitor Irosustat when added to an aromatase inhibitor in ER-positive breast cancer patients
Source: Breast Cancer Res Treat. 2017 Jun 13;165(2):343–53. doi: 10.1007/s10549-017-4328-z (PMC5543190; doi:10.1007/s10549-017-4328-z)
Supplement: Supplementary file 3 — Supplementary material 3 (DOCX 19 kb) [file 10549_2017_4328_MOESM3_ESM.docx]

| **Patient ID** | **Sample type** | **STS**  **(P + IS)** | **EST**  **(P + IS)** | **AROM^†^**  **(P + IS)** | **17BHSD1**  **(P + IS)** | **17BHSD2**  **(P + IS)** | **Clinical Benefit by sites' review** | **Clinical Benefit by central review** |
| --- | --- | --- | --- | --- | --- | --- | --- | --- |
| IRIS01-003 | Diagnostic biopsy | 2  (1+1) | 4  (2+2) | 4  (2+2) | 3  (2+1) | 3  (2+1) | No | No |
| IRIS01-006 | Diagnostic biopsy | 4  (2+2) | 5  (2+3) | 5  (3+2) | 4  (2+2) | 5  (2+3) | No | No |
| IRIS01-007 | Surgical excision | 5  (2+3) | 4  (2+2) | 6  (3+3) | 4  (2+2) | 3  (2+1) | No | No |
| IRIS01-009 | Diagnostic biopsy | 3  (2+1) | 4  (2+2) | 5  (3+2) | 3  (2+1) | 4  (2+2) | No | No |
| IRIS02-001 | Surgical excision | 3  (2+1) | 4  (2+2) | 6  (3+3) | 4  (2+2) | 3  (2+1) | No | No |
| IRIS02-002 | Surgical excision | 3  (2+1) | 4  (2+2) | 3  (2+1) | 3  (2+1) | 2  (1+1) | No | No |
| IRIS02-003 | Surgical excision | 3  (2+1) | 4  (2+2) | 5  (3+2) | 4  (2+2) | 4  (2+2) | No | No |
| IRIS02-004 | Surgical excision | 2  (1+1) | 4  (2+2) | 6  (3+3) | 3  (2+1) | 2  (1+1) | No | No |
| IRIS03-001 | Surgical excision | 3  (2+1) | 3  (2+1) | 6  (3+3) | 4  (2+2) | 3  (2+1) | Yes | No |
|  | 1st relapse | 2  (1+1) | 3  (2+1) | 2  (1+1) | 2  (1+1) | 2  (1+1) | Yes | No |
| IRIS04-001 | Progression | 2  (1+1) | 4  (2+2) | 6  (3+3) | 3  (2+1) | 2  (1+1) | No | Yes |
| IRIS04-002 | Diagnostic biopsy | 3  (2+1) | 3  (2+1) | 4  (2+2) | 4  (2+2) | 2  (1+1) | No | No |
| IRIS06-001 | Diagnostic biopsy | 5  (2+3) | 4  (2+2) | 6  (3+3) | 4  (2+2) | 3  (2+1) | No | No |
| IRIS06-002 | Surgical excision | 2  (1+1) | 3  (2+1) | 5  (3+2) | 4  (2+2) | 4  (2+2) | No | No |
|  | 1st relapse | 3  (1+2) | 5  (2+3) | 6  (3+3) | 5  (2+3) | 2  (1+1) | No | No |
| IRIS06-003 | Surgical excision | 5  (2+3) | 4  (2+2) | 6  (3+3) | 3  (2+1) | 2  (1+1) | No | No |
|  | 1^st^ Relapse | 5  (2+3) | 5  (2+3) | 6  (3+3) | 4  (2+2) | 5  (2+3) | No | No |
| IRIS06-004 | Diagnostic biopsy | 5  (2+3) | 4  (2+2) | 5  (3+2) | 4  (2+2) | 4  (2+2) | No | No |
| IRIS06-005 | Diagnostic biopsy | 3  (1+3) | 3  (2+1) | 5  (2+3) | 3  (2+1) | 3  (1+2) | No | No |
| IRIS10-001 | Surgical excision | 4  (2+2) | 4  (2+2) | 5  (3+2) | 3  (2+1) | 4  (2+2) | Yes | Yes |
| IRIS10-003 | Surgical excision | 4  (2+2) | 3  (2+1) | 6  (3+3) | 4  (2+2) | 2  (1+1) | No | No |
| IRIS10-004 | Diagnostic biopsy | 2  (1+1) | 4  (2+2) | 3  (2+1) | 4  (2+2) | 2  (1+1) | No | No |
| IRIS10-005 | Diagnostic biopsy | 3  (2+1) | 3  (2+1) | 6  (3+3) | 4  (2+2) | 3  (2+1) | Yes | No |
| IRIS01-005^‡^ | 1st relapse | - | - | - | - | - | Yes | Yes |
| IRIS01-001^**^ |  |  |  |  |  |  | Yes | Yes |
| IRIS01-002^**^ |  |  |  |  |  |  | No | No |
| IRIS01-004^**^ |  |  |  |  |  |  | No | No |
| IRIS01-008^**^ |  |  |  |  |  |  | No | No |
| IRIS01-010^**^ |  |  |  |  |  |  | No | No |
| IRIS04-003^**^ |  |  |  |  |  |  | No | No |

**Table S2 Summary of immunohistochemistry staining scores for steroidogenic enzymes**

**Arom: aromatase, EST:estrogen sulfotransferase;STS: steroid sulfatase;** **17BHSD1: 17beta-Hydroxysteroid dehydrogenase type 1; 17BHSD2: 17beta-Hydroxysteroid dehydrogenase type 2**

Scoring for proportion (P) except AROM: 0: no stained tumour cells; 1: 1–50%; 2: >50% immuno-positive cells;

† Scoring for proportion (P) of AROM: 0: <1%; 1: 1–25%, 2: 26–50%, and 3: >50% immuno-positive cells;

Scoring for intensity (IS): 0: No stained tumour cells; 1: Weak staining; 2: Moderate staining; 3: Strong staining;

‡ No stained tumour cells

** No samples were collected for steroidogenic enzyme tests
