# Supplementary figures and images for: IRIS study: a phase II study of the steroid sulfatase inhibitor Irosustat when added to an aromatase inhibitor in ER-positive breast cancer patients
Source: Breast Cancer Res Treat. 2017 Jun 13;165(2):343–53. doi: 10.1007/s10549-017-4328-z (PMC5543190; doi:10.1007/s10549-017-4328-z)

## Slide 1
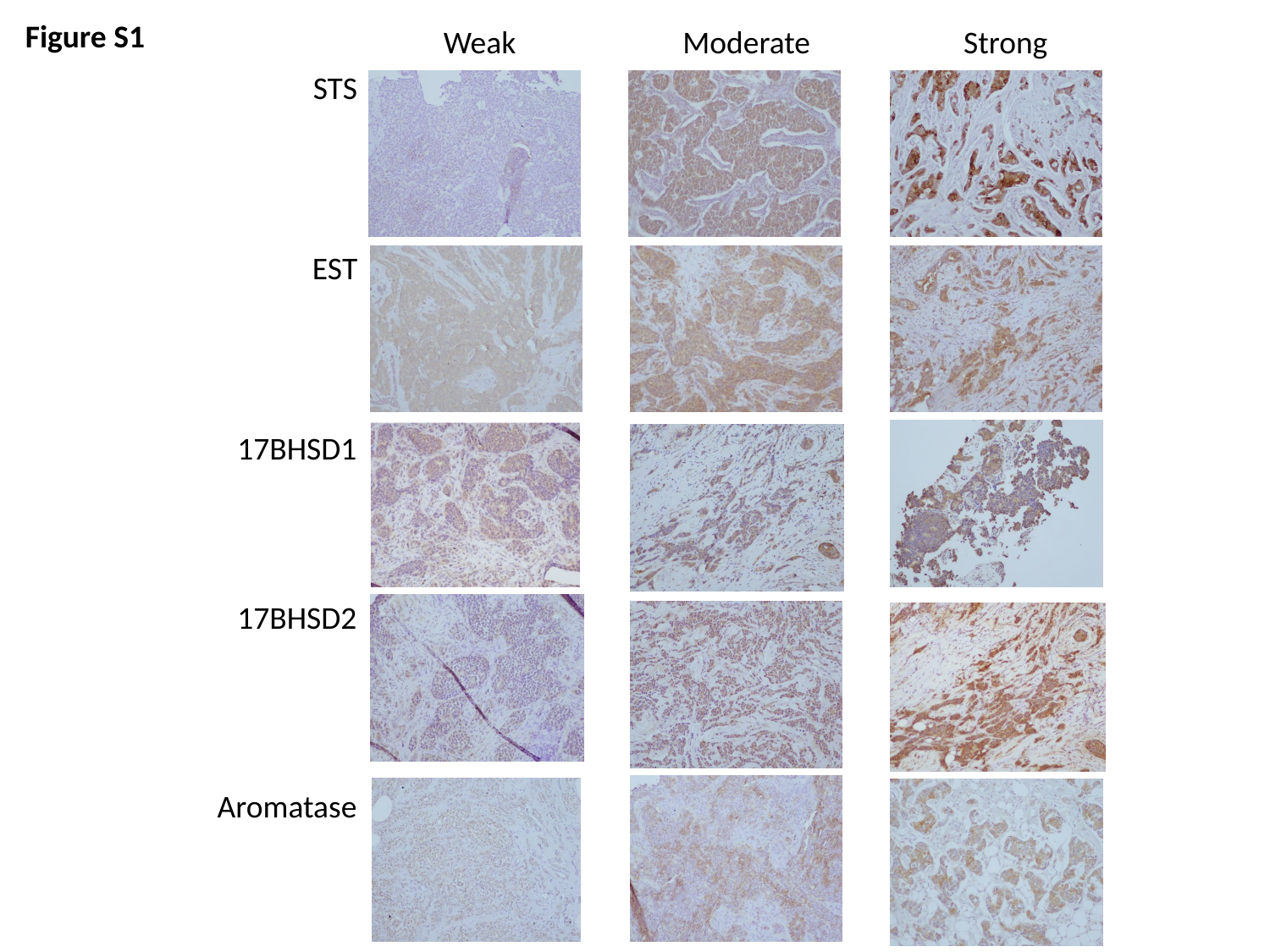

Figure S1
Weak
Moderate
Strong
STS
EST
17BHSD1
17BHSD2
Aromatase

Supplement: Supplementary file 4 — Supplementary material 4 (PPTX 22879 kb) [file 10549_2017_4328_MOESM4_ESM.pptx]
